# The Key Element Role of Metallophores in the Pathogenicity and Virulence of *Staphylococcus aureus*: A Review

**DOI:** 10.3390/biology11101525

**Published:** 2022-10-18

**Authors:** Ghassan Ghssein, Zeinab Ezzeddine

**Affiliations:** Department of Laboratory Sciences, Faculty of Public Health, Islamic University of Lebanon, Khalde P.O. Box 30014, Lebanon

**Keywords:** metallophores, virulence, *Staphylococcus aureus*, siderophores, staphylopine, metal ions

## Abstract

**Simple Summary:**

Metallophores, which are secondary metabolites secreted by bacteria, play an important role in their virulence. The bacterium *Staphylococcus aureus* produces several types of metallophores, such as staphyloferrin A, staphyloferrin B, and staphylopine, which are responsible for metal ion sequestering. The former is specific for iron chelating, while the latter is a wide-spectrum metallophore, but it mainly chelates zinc. The detailed description of the biosynthesis, export and import processes of each metallophore type is highlighted in this review. Moreover, the genetic regulation is explained as well. Previous studies that provide evidence about the crucial part of metallophores in *Staphylococcus aureus* pathogenesis were also mentioned herein.

**Abstract:**

The ubiquitous bacterium *Staphylococcus aureus* causes many diseases that sometimes can be fatal due to its high pathogenicity. The latter is caused by the ability of this pathogen to secrete secondary metabolites, enabling it to colonize inside the host causing infection through various processes. Metallophores are secondary metabolites that enable bacteria to sequester metal ions from the surrounding environment since the availability of metal ions is crucial for bacterial metabolism and virulence. The uptake of iron and other metal ions such as nickel and zinc is one of these essential mechanisms that gives this germ its virulence properties and allow it to overcome the host immune system. Additionally, extensive interactions occur between this pathogen and other bacteria as they compete for resources. *Staphylococcus aureus* has high-affinity metal import pathways including metal ions acquisition, recruitment and metal–chelate complex import. These characteristics give this bacterium the ability to intake metallophores synthesized by other bacteria, thus enabling it to compete with other microorganisms for the limited nutrients. In scarce host conditions, free metal ions are extremely low because they are confined to storage and metabolic molecules, so metal ions are sequestered by metallophores produced by this bacterium. Both siderophores (iron chelating molecules) and staphylopine (wide- spectrum metallophore) are secreted by *Staphylococcus aureus* giving it infectious properties. The genetic regulation of the synthesis and export together with the import of metal loaded metallophores are well established and are all covered in this review.

## 1. Introduction

*Staphylococcus aureus* (*S. aureus*) is considered one of the most widespread infectious bacteria. It is found in the environment as well as being part of the human skin and nasal microbiota [1]. Normally, *S. aureus* is harmless on healthy skin, but once it enters the blood or internal tissues, diverse infections occur including pneumonia, infection of surgical site and nosocomial bacteremia [2]. Systemic *S. aureus* infection depends on the bacteria breaking through the epithelial protective layer. The incidence rate of this serious medical condition is between 20 and 50 cases/100,000 per year, with fatality rate ranging from 10% to 30% [3]. Moreover, *S. aureus* forms biofilms that are associated with medical device infections such as prosthetic joints and endocarditis [4]. The prevalence of antibiotic resistance *S. aureus* isolates, methicillin-resistant *S. aureus* (MRSA), is posing a serious problem for combating infectious diseases caused by this pathogen [5].

This bacterium is Gram-positive, its cell wall composed of a lipid membrane surrounded by a thick peptidoglycan and teichoic and lipoteichoic acid layer [6]. The rigidity of the cell wall is determined by these peptidoglycan chains that gives the bacterium its shape and protects it from osmotic lysis [7]. The staphylococcal cell surface is negatively charged due to the presence of teichoic acids, which contain phosphate and play a role in the localization and acquisition of metal ions [8]. *S. aureus* produces notable virulence factors, contrary to some other bacteria that promote disease only by secreting toxins. These factors include an excessive amount of immune-evasion substances and a wide range of small-molecule mediators, also called secondary metabolites, that enable host colonization during infection and give *S. aureus* the ability of successful competition with other microorganisms in nutrient-poor conditions. The biosynthesis of such metabolites involves complex enzymatic cascades, and they are considered vital components of staphylococcal interactions, with microbiome occupying the same niche e.g., human nares [9]. Such mediators belong to a large variety of protein and non-protein compounds that have attractive properties for future drug development. Examples of these molecules include bacteriocins (lanthipeptides, thiopeptides, fibupeptides) that inhibit bacterial competitor species; signaling molecules such as thiolactone peptides that induce or inhibit sensory cascades in other bacteria; or metallophores (staphyloferrins and staphylopine) that are transition metal ions binding molecule [10].

This review focuses on metallophores due to their crucial role during both infections and bacteria–microbiome interactions. These low-molecular-weight molecules are produced by bacteria during scant availability of essential transition metal ions, like iron or zinc. In *S. aureus*, metal ion cofactors are required in various biochemical processes such as nucleic acid and protein synthesis, virulence factor expression regulation, DNA replication and reactive oxidative species (ROS) metabolism [11]. Metallophores can import metal ions into the bacteria even when the amount in the surrounding environment is extremely low. This enables the invading bacterium to obtain essential metallic nutrients from the human body and endure “nutritional immunity” even when the immune system is acting in full force to limit the availability of metal ions [12].

Metal transport systems of *S. aureus* are essential for its virulence, especially those required for iron and manganese accretion [13]. The uptake of iron can be conducted by the secretion of siderophores that form soluble Fe^3+^ complexes in order to be actively taken up via specific receptors [14]. Four siderophores synthesized and secreted by *S. aureus* were previously described in the literature. The most studied are the two high-affinity siderophores (staphyloferrin A and staphyloferrin B) that bind to free iron and compete with host iron sequestering proteins lactoferrin and transferrin [15]. Then HtsABC and SirABC transporters import iron bound to staphyloferrin A and B, respectively [16]. However, *S. aureus* has metal requirements other than iron. This pathogen also produces staphylopine, which is another metal chelator that allows it to uptake several metals, including nickel and zinc, from the host during infection [17]. In previous studies, an ABC metal transport system (ATP-binding cassette) named CntABC (Cnt, cobalt-nickel transporter) was identified in *S. aureus* [18]. This ABC transporter allows *S. aureus* to internalize nickel and cobalt under low-zinc conditions through CntA (solute-binding protein) and contributed to *S. aureus* virulence of urinary tract infection in mice [19]. In addition, it produces hemolysins that rupture red blood cells membranes causing hemoglobin release which is then degraded to heme and free iron by a series of processing and import steps brought about by the Iron-Regulated Surface Determinant (Isd) staphylococcal system [20].

The presence of metalloregulators (metal-dependent regulatory proteins) in *S. aureus* as in other organisms allows it to sense the metals’ bioavailability and maintain metal ion homeostasis. This metal regulation is achieved through the transcription control of the genes involved in metal ion import, storage, distribution and efflux. The metal-sensing mechanism requires the reversible interaction of one or more specific metal ions with the regulator protein, so that the affinity of the regulatory protein for the specific DNA sequences located in target promoter operons will be altered. Such alterations in the metalloregulator occupation on the operator regulate target gene transcription [21]. These processes maintain suitable transition metal ion levels, thus allowing *S. aureus* adjustment to changing host environment conditions and shifting metabolic demands.

## 2. Metallophores Produced by *Staphylococcus aureus*

### 2.1. Siderophores

Iron, which has multiple oxidative states, is needed in many vital life processes such as electron transfer and DNA replication [22]. Bacteria require iron as an enzyme cofactor in the catalysis of redox reactions included in their basic cellular processes [23]. In the human body the level of free iron ions is extremely low since most iron is confined to storage, metabolic molecules and transport. Ferrous Fe^2+^ ions are exceedingly toxic due to their association with the Fenton reaction that produces harmful hydroxyl radicals [24]. On the other hand, ferric Fe^3+^ ions are insoluble at physiological pH and thus not readily bioavailable [25]. In order to endure this iron shortage iron, bacteria developed several mechanisms for obtaining iron from the host since it is important for their colonization during infection. These mechanisms include acquiring heme-bound iron, absorption by membrane-bound intake systems and siderophore secretion [26]. Siderophores are iron high-affinity metallophores and are essential pathogenicity factors in bacteria including *S. aureus.* The latter produces and secretes two staphyloferrins (siderophores) into the extracellular environment to scavenge iron. In addition, *S. aureus* has specific uptake systems for these staphyloferrins and for siderophores produced by other microorganisms as well [27].

There are four distinguished types of siderophores, catecholate, phenolate, hydroxamate and carboxylate, classified according to their iron chelation moieties [28,29]. The synthesis of siderophores is achieved either by non-ribosomal peptide synthesis (NRPS) or by NRPS-independent siderophore (NIS) synthesis (polyketide synthase (PKS) domains) that function together with NRPS units [30]. Also, a small quantity of siderophores is produced independent of these two pathways [31]. The NRPS pathway is the most common while that of NIS is less characterized.

NRPS siderophores have peptidic scaffolds, often incorporating nonproteinogenic amino acids and their derivatives, which are assembled stepwise with covalently bound intermediates [32]. In the NIS pathway, the covalent attachment of intermediates to the enzymes was not noted [30]. In the first synthesis route, siderophores are manufactured by the assembly of individual enzymes where dicarboxylic acids are condensed with diamines, amino alcohols, and alcohols in alternating subunits. Further subunit modifications (decarboxylation, oxidation or isomerization) are performed by distinct enzymes encoded by clusters of genes located near those related to synthetases encoding. Composite pathways using both assembly types were also reported [33]. *S. aureus* uses the NIS pathway in the synthesis of its staphyloferrins. Siderophores secretion is an active process (energy driven) and is flowed out through transport pumps [28].

The intake of iron chelated by siderophores varies between Gram-negative and Gram-positive bacteria due to the presence of an outer membrane in Gram-negative bacteria through which they should be transported [34,35]. In Gram-negative bacteria, the loaded siderophores are recognized specifically by receptors (β-barrel) found in the outer membrane. The change in the receptors conformation once the ligand is bound allows the translocation of loaded siderophores into the periplasm. [36]. Then, the transport into the cytoplasm is mediated by an ABC transporter located in the inner membrane [37]. The iron is reduced in the periplasm in some cases, and only Fe^2+^ ion is brought into the cytosol [38]. Concerning Gram-positive bacteria, the import of siderophores is directly achieved by an ABC transporter extending across the cell membrane because there is no outer-membrane receptors [39]. After iron release, siderophores may be either recycled [40] or hydrolyzed [41].

#### 2.1.1. Ferric Uptake Regulator (Fur)

In the host iron depleted environment, *S. aureus* alters its profile of protein expression. Such change is conveyed by the iron-dependent ferric uptake regulator (Fur) [42]. When iron is present, Fur binds to a DNA consensus sequence noted as the Fur box located upstream the Fur-regulated genes and stops their transcription. On the contrary, when iron is scarce, Fur is released from the DNA, reducing Fur-mediated transcriptional suppression. [43]. The imaging of mice infected with *S. aureus* revealed the expression of Fur-regulated genes in abscesses found in heart and kidneys, which suggested that the bacterium was iron starved in these organs [44,45]. Upon comparing the protein profiles in the cytoplasm of wild type *S. aureus* and Fur mutant type, twenty staphylococcal proteins were found to be more plentiful when Fur was absent, indicating that Fur negatively regulates these proteins [13]. Furthermore, this investigation showed increases in fermentative and glycolytic enzymes, demonstrating that *S. aureus* regulates its metabolism so that it can adjust to iron-scant host environment. This lactate secretion decreases the microenvironment pH and thus the transferrin affinity for iron [46]. These data reveal that the host environment is altered by *S. aureus* so that iron is released from the host proteins assuming the increase in its bioavailability. Fur also regulates the virulence factors expression involved in host cell attachment and formation of biofilm [47,48].

Fur is considered a suppressor that uses Fe^2+^, it prevents transcription when iron is available through binding to the siderophores operon promoter region. This suppression stops when iron concentration decreases and siderophores biosynthesis and transport genes are allowed [49]. Three Fur homologues (Fur, PerR, and Zur) are present in *S. aureus*. Fur represses both the hydroxamate intake system fhuD2 genes and the sirABC and sstABCD transport operons for siderophores whenever iron is available. On the other hand, the storage of iron and the resistance of oxidative stress are regulated by PerR. The latter expresses variable genes whenever iron is available such *kat*A, *ahp*CF, *bcp*, *trx*B, *ftn*, which encode catalase, alkyl hydroperoxide reductase, bacterioferritin comigratory protein, thioredoxin reductase and ferritin respectively [50]. Also, PerR was shown to be important for *S. aureus* virulence in mice. Concerning Zur, it is responsible for zinc regulation.

#### 2.1.2. Staphyloferrin A

This highly hydrophilic, carboxylate-type siderophore (480 Da) is fabricated by the condensation of two citrate molecules to a D-ornithine backbone [51]. The products of *sfaABCD* operon regulated by Fur are responsible for both the synthesis and export of Staphyloferrin A [52]. In *S. aureus*, the two synthetases SfaD and SfaB, which belong to the NIS class and encoded by its genome, perform the catalysis of condensation reactions producing apo- Staphyloferrin A. These reactions proceed when SfaD form an amide bond between first citrate and D-ornithine δ-amine by using it as a nucleophile. Then, SfaB catalyzes the formation of a new bond between the second citrate and the D-ornithine δ -amine through nucleophilic substitution [53]. Staphyloferrin A is exported and carried out from the cell by SfaA that is analogous to known efflux transmembrane proteins and belongs to the principal facilitator superfamily [54]. The production of this siderophore depends on its precursor’s availability. It is supposed that SfaC, a recognized racemase, converts D-ornithine from L-ornithine found in the cytoplasm [55]. The availability of citrate is temporary, where TCA cycle (Tricarboxylic Acid Cycle) is needed for Staphyloferrin A production in vivo [56].

The import of iron Fe^3+^ loaded Staphyloferrin A is arbitrated by the heme transport system ABC transporter denoted as HtsABC. [52]. Fur regulates the operon of *htsABC* which is located next to the Staphyloferrin A biosynthesis *operon sfa*. HtsA is spontaneous bacterial peritonitis (SBP) and HtsBC is the heterodimeric permease integrated in the membrane. The Hts system depends on FhuC (an unselective ATPase) because the ATP-binding protein gene is absent in *htsABC operon*. On the other hand, *S. aureus* has *ntrA* gene, regulated by Fur, that encodes a nitroreductase needed for iron loaded Staphyloferrin A utilization [57].

This bacterium does not produce glutathione but uses bacillithiol instead for its redox homeostasis [58]. This metabolite might have a comparable function with NtrA. Moreover, it was suggested that NtrA is essential for Fe^3+^-Staphyloferrin A usage since *ntrA* mutant strain, lacking Staphyloferrin B as well, had reduced growth in iron-depleted media [57]. Contrary, NtrA is not needed for Fe^3+^- Staphyloferrin B or Fe^3+^-desferrioxamine utilization proposing a unidirectional iron transfer from desferrioxamine to Staphyloferrin A and then released by NtrA [57].

#### 2.1.3. Staphyloferrin B

Staphyloferrin B (448 Da) is also a carboxylate-type siderophore. Its biosynthesis and export is mediated by enzymes encoded by Fur regulated gene cluster *sbn* (*sbnABCDEFGHI*) [59]. This siderophore is assembled from a molecule of citrate and another of α-ketoglutarate with two molecules of L-Dap (L-2,3-diamopropionic acid). Such assembly is achieved by SbnC, SbnE, SbnF, which are NIS-synthetases, and SbnH (a decarboxylase) [60]. Contrary to the Staphyloferrin A, the gene cluster *sbn* encodes enzymes that synthesize the three precursors of Staphyloferrin B, in addition to the proteins responsible for its assembly [61]. Citrate is produced by SbnG, while SbnA and SbnB work in conjunction in order to produce the two building blocks (α-ketoglutarate and L-Dap) needed for the synthesis of Staphyloferrin B [62]. Concerning *sbnI*, the cluster last gene, it has a role both in the regulation and the precursor biosynthesis. This gene is needed for *sbnD-H* full expression, consequently regulating in vivo iron acquiring by Staphyloferrin B [63]. Furthermore, SbnI generates SbnA substrate [64]. Staphyloferrin B is produced by *S. aureus* in a wide range of mediums, for example infections of bones and blood, which differ in nutrient availability (oxygen and glucose), so the bacterium metabolism will be altered. Thus, Staphyloferrin B can be produced by SbnA, B, G, and I apart from the TCA cycle or glycolysis [64].

When the assembly is complete, the export from the cell is mediated by SbnD [59,60]. The latter is a facilitator protein having ten transmembrane domains. However, there exist additional exporters, which is not the case for Staphyloferrin A [54]. It was found that the extracellular levels of Staphyloferrin B decreased while the intracellular levels increased in *sbnD S. aureus* deletion strain after 14 h under iron depleted growth conditions. However, after a lag period of 36 h, the extracellular and intracellular levels were similar to those in wild-type bacterium, indicating the activation of an exporter independent of SbnD [54].

*S. aureus* takes iron-loaded Staphyloferrin B by the staphylococcal iron regulated system (Sir), encoded by the operon *sirABC*, which is regulated by Fur [16]. The disruption of SirA (lipoprotein for substrate binding) or SirB (membrane permease) causes weakened growth in iron-confined conditions [16]. Similar to the Hts system, the FhuC ATPase connects with SirABC forming a complete ABC transporter [65]. The iron release mechanism from Staphyloferrin B when it reaches the cytoplasm is still not known.

The binding orientations for SirA and HtsA are so different since only few siderophore binding residues are conserved between them despite of the resemblance in their structure. Tthe specificity of SirA for iron loaded Staphyloferrin B and HtsA for iron loaded Staphyloferrin A, is attributed to these differences in the residues responsible for each siderophore binding [66].

**Figure 1:**
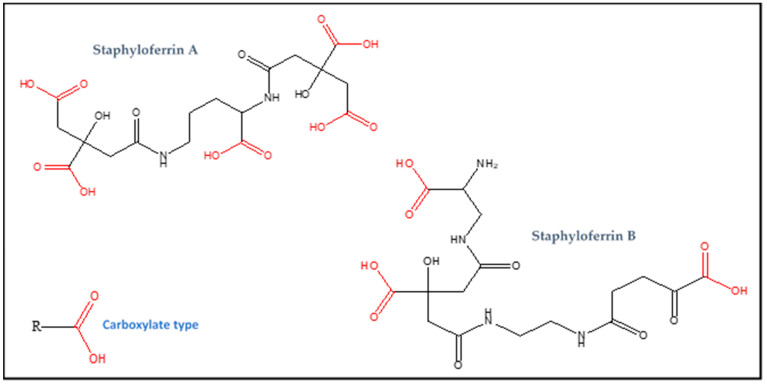
The structure of the two siderophores: Staphyloferrin A and B.

The structures of Staphyloferrin A and Staphyloferrin B are illustrated in Figure 1.

#### 2.1.4. Staphylobactin

This siderophore was discovered upon studying the virulence of siderophores in *S. aureus*. In an infected mouse model, the siderophore-producing strains revealed higher virulence than those that lacked siderophores [59]. The operon responsible for the siderophore production in the study was named *sbn* as well. The researchers found that *sbn*E mutants were avirulent and termed the siderophore extracted staphylobactin; they later found that it is a hydroxamate type having citric acid residues [67]. Like other *S. aureus* siderophores, staphylobactin was transported via ABC transporter. *sir*B or *sir*A mutants were not able to uptake Fe-staphylobactin [59].

#### 2.1.5. Aureochelin

Aureochelin (577 Da) is another siderophore produced by *S. aureus.* Its structure is still unknown, but it might be a phenolate catecholate type due to its reactivity with the Swain reagent that is used to detect phenolic moieties. Two surface-associated proteins of molecular weight 120 and 88 kDa were found to be expressed in iron depleted conditions and were related with the production of Aureochelin. Moreover, these proteins were also detected as antigenic [15].

#### 2.1.6. Xenosiderophores Uptake by *S. aureus*

*S. aureus* is able to uptake siderophores produced by other microorganisms and it has both hydroxamate and catechol exogenous siderophores uptake systems thus enabling it to compete with other bacteria. Concerning the hydroxamate system, it is moderated by an ABC transporter named Fhu (ferric hydroxamate uptake) system and consists of the operon *fhuCBG* and FhuD1 and FhuD2 proteins regulated by Fur [68]. FhuB and FhuG are very hydrophobic and bound to the membrane, while an ATP-binding protein is coded by *fhu*C [69]. The genes *fhuD1* and *fhuD2* are outside the operon *fhuCBG* and code for lipoprotein receptors [70]. FhuD2 has a wider hydroxamate range and higher affinity than FhuD1 [68]. The import of exogenous hydroxamate siderophores ferrichrome, aerobactin, ferrioxamine B and coprogen involves FhuD2. On the other hand, FhuD1 transports only ferrioxamine B and ferrichrome. Unlike other known transport systems, no conformational changes were shown for FhuD2 during iron-hydroxamate binding [71].

The staphylococcal siderophore transporter (*sstABCD*) operon encodes the ABC transporter responsible for iron-catechol siderophores uptake. Heterodimeric permease is formed by SstA and SstB. SstC is an ATP-binding homodimeric protein, and SstD is the lipid-anchored substrate-binding proteins (SBP) [72,73]. *SstD* is expressed mostly in iron depleted conditions, [72]. The uptake of ferric enterobactin, 2,3-dihydroxybenzoic acid (DHBA) and bacillibactin complexes strictly requires SstABCD. Although the Sst system can import also salmochelin 4 and petrobactin, there exists another transporter involved in their in vivo uptake, but it is still unidentified [51]. Furthermore, the catecholamine- iron complexes import requires SstABCD. Epinephrine, for example, which is a catecholamine hormone, is known to reduce iron bound to transferrin thus forming iron complexes that *S. aureus* can use as a source of iron [74].

#### 2.1.7. The Virulence of Staphylococcal Siderophores

Greater siderophore production in *S. aureus* is associated with its virulence manifested through additional bacterial counts, larger abscesses and further inflammation in infected mice [75]. Moreover, the strains that showed higher resistance to the neutrophils in vitro were found to produce more siderophores [75]. Mutant types that were unable to produce staphylobactin could not persevere in mice. This finding suggests that uptake of iron by sources other than non-siderophores might be necessary in the early infection stages, while siderophores are essential in later infection stages. The deletion of the chromosomal *sbn* operon impaired the growth of *S. aureus* in serum [59], emphasizing the significance of iron uptake schemes for the pathogenesis of this bacterium.

### 2.2. Additional Metal Acquiring Systems

In addition to the iron uptake system, *S. aureus* have other various systems of transportation for transitional metal ions, such as Cnt, Adc, NixA and Nik [18,76,77]. The latter is an ABC transporter and is essential for bacterial acquisition of nickel. This system (Nik) is effective in delivering nickel by the means of small chelating molecules (e.g., L-histidine), determining the activity of urease, and having a crucial role in the mouse urinary tract colonization [78,79]. Another nickel-acquiring system in *S.* *aureus* is NixA, which is a secondary transporter of NiCoT (nickel-cobalt transporter) membrane protein family. Along with Nik, NixA is also critical for the activity of urease and colonization in kidney [19]. Adc system is responsible for zinc uptake in Gram-positive bacteria including *S. aureus* and it is composed of AdcA that is a metal acquiring unit and AdcBC which is an ABC transporter [17]. Cnt, on the other hand, can transport several metals such as nickel, zinc, copper, cobalt, zinc, and manganese [17] but at zinc scarce conditions, it serves as a zinc uptake system [17].

#### 2.2.1. Metallophore Staphylopine

**Figure 2:**
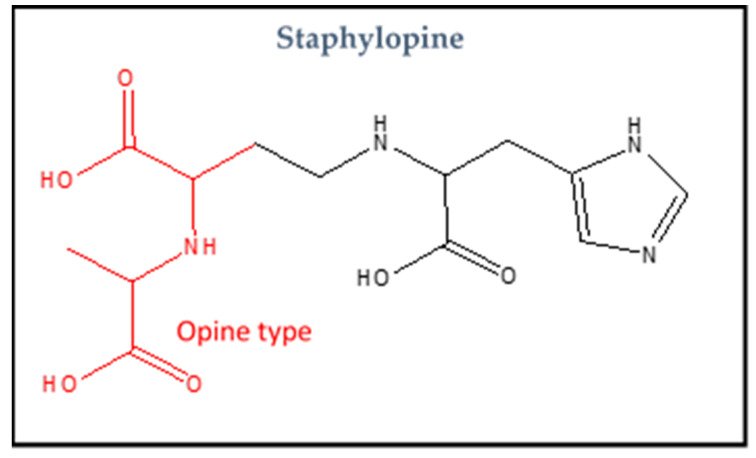
The structure of metallophore staphylopine.

In the first place, *S. aureus* utilizes Adc for importing zinc. Cnt system will be aroused when Adc alone becomes unable to meet zinc cellular requirement. A distinctive characteristic of Cnt is utilizing staphylopine, which is a nicotianamine-like metallophore [17]. This last contains imidazole ring and three carboxylic groups. It is an opine metallophore and can chelate several metal ions (nickel, zinc, cobalt, iron and copper), so it is considered a broad-spectrum metallophore [80]. The import of these wide range of metal ions via staphylopine depends on the metal nature and concentration along with the *S. aureus* growth status [79]. The structure of staphylopine is shown in Figure 2.

#### 2.2.2. Staphylopine Synthesis

Nine genes in the operon *cnt (cntKLMABCDFE)* encodes the multiple functions needed for the synthesis and the exportof staphylopine in addition to the import of the complexe (Staphylopine-metal ion). The three genes, *cntKLM*, encode the needed enzymes for staphylopine biosynthesis CntK, CntL and CntM respectively. The five genes *cntABCDF* encode the transporter ABC implicated in metal loaded staphylopine import [81,82]. Concerning *cntE*, it is involved in staphylopine export by encoding transport protein located in the bacterium membrane. All *Cnt* genes are most expressed in metal scant medium [80]. The importer protein CntA plays a main role in initiation of metal loaded staphylopine recognition and importation [18,78,79]. The mechanism of recognition and transportation of metal loaded staphylopine at the molecular level was verified by the interdomain change that occurs in CntA conformation upon binding to metal loaded staphylopine loaded [83]. The two CntB and CntC proteins located in membrane form a channel that may have a role in staphylopine loaded transportation [18,79,80]. As for ATP-binding CntD and CntF membrane proteins, they supply the energy needed for transportation [18,78]. The uptake of iron, nickel, zinc and cobalt decreases in *S. aureus cntL* and *cntA-F* mutant strains [79]. Zinc represses the transcription from the promoter *cntA* [18]. *S. aureus* also has Zur which represses the operon that encodes the two proteins related to ABC transporter [84]. If iron is available, Fur represses *cnt* genes [82]. These data designate that the expression of *cnt* gene is limited in zinc and iron rich environment and that *Cnt* system is controlled by both Fur and Zur [85] so, the synthesis of staphylopine is under negative control by Fur/Zur binding. On the other hand, staphylopine export and staphylopine metal recovery is less repressed by cooperative Fur/Zur repression [85].

Three steps are involved in the biosynthesis of staphylopine [80]. Firstly, D-histidine is produced via CntK which is a histidine racemase. Then, the enzyme CntL, that resembles nicotianamine-synthase, uses D-histidine as a substrate and catalyzes the production of xNA (the name comes from its nicotianamine correlation). The latter in an intermediate that is produced through the addition of aminobutyrate (an S-adenosyl methionine moiety). The last step involves the enzyme CntM that condensates pyruvate with xNA producing staphylopine [79]. CntM has the biochemical characteristics of opine synthase enzyme members [79]. A study done in vitro has found that metals employ several effects on the CntM catalyzed reaction [86]. They noticed that at low concentration of copper and zinc, the reaction was moderately activated but totally inhibited at high concentration. Manganese, on the other hand, was an activator only while nickel and cobalt were inhibitors only so it was proposed that the metal affinity toward xNA and an enzyme inhibitory binding site controlled the activation or inhibition according to the concentration of metals. This regulation of the enzyme involved in staphylopine synthesis is dependent on metal may happen in vivo as well and can could help in the adjustment of the production of metallophore [86].

Table 1 summarizes the properties of each *S. aureus* metallophore mentioned in in this review and Table 2 summarizes their regulation and transportation.

#### 2.2.3. Staphylopine as Zincophore

Nutritional immunity drastically limits the bioavailability of zinc during bacterial infection [87]. In spite of this essential nutrient restriction, *S. aureus* remains capable of causing severe disease because it is able to compete for zinc with the host [88]. As previously mentioned, *S. aureus* has two distinct ABC permease types involved in zinc acquisition, AdcABC and CntABCDF. AdcABC is homologous to ABC permeases associated with direct zinc recruitment, while CntABCDF belongs to the NikA/Opp family of ABC permeases. CntABCDF functions in conjunction with staphylopine to specifically promote zinc acquisition. This indicates that staphylopine functions as a staphylococcal zincophore although it can bind various metals in vitro. In a study performed by Grim et al. [17], they found that in zinc depleted medium, strains lacking the Cnt-staphylopine system and Adc permease had major growth defects and failed specifically in zinc accumulation. These results demonstrated that both systems serve as the major zinc importers of this bacterium. Concerning other metal ions such as Co, Ni, and Cu, they found that they are not physiological substrates of the Cnt-staphylopine system, and it is modestly responsive to Mn and Fe [82] which only exert transcriptional influence in the absence of zinc. These findings suggest that the abundance of zinc is the main regulatory factor that controls the system expression [17].

#### 2.2.4. Important Features of Cnt-Staphylopine System

The Cnt system, as previously mentioned, is essential for the optimal metals import metal-limiting conditions and contributes to *S. aureus* virulence. The failure to efflux staphylopine results in its intracellular accumulation thus impairing the fitness of *S. aureus* [89]. A recent study has shown that CntE loss resulted in a stronger virulence defect than other components of the Cnt-staphylopine system, even in zinc restricted tissues. The toxicity associated with intracellular staphylopineaccumulation contributed to the virulence defect of strains lacking CntE, even when *S. aureus* is zinc starved during infection. Moreover, they noticed that the intracellular accumulation of staphylopine did not increase metal importer expression or altered cellular metal concentrations, suggesting that contrary to prevailing models, the toxicity associated with staphylopine is not strictly due to intracellular chelation of metals [90]. CntK catalyzes the first step of staphylopine synthesis by converting L -histidine to D -histidine in order to provide an essential building block of staphylopine. It was found, by structural modeling, that CntK is specific for histidine, whereas other proteinogenic amino acids, with the exception of arginine, do not show any binding with it. These findings helped in developing powerful antibiotics targeting the staphylopine-mediated metal acquisition process in bacteria via designing irreversible inhibitors [91]. Another study confirmed that during the synthesis of staphylopine, CntL stereoselectively carries out the catalysis of D-histidine and not L-histidine. These findings provided critical structural and mechanistic insights into CntL for a better understanding of of nicotianamine-like metallophores biosynthesis and the discovery of inhibitors of this process [92]. Concerning CntA, responsible for the recognition and transport of diverse solutes, a study was performed to investigate the structural conformation upon staphylopine binding. CntA has a fork-like structure formed by three domains (I_a_ and I_b_ and II). It uses a bi-domain architectural form of domain II assisted by inter-domain hinge cluster residues. Important clustered communities regulat the conformational changes in CntA. In addition to open (without staphylopine) and close states (with staphylopine) [83], the fluctuating regions sampled two additional intermediate states that were considered closed or open previously. CntA prefers fluctuating the non-conserved regions rather than conserved where domain II turned out to be rigid and maintains a stable fold. Such findings are important to the researcher in field of drug-designing [93].

As for the regulation of cnt operon, a novel regulator (Rsp) was identified that activates the system, in addition to the metal-dependent Fur and Zur repressors. This regulator is an AraC-type regulator. Rsp activation in *S. aureus* may act to maintain basal cellular levels of staphylopine to scavenge free metals when needed [94]. It is worth mentioning that the AraC family regulators are an abundant group of transcriptional regulators in bacteria, acting mostly as gene expression activators, that controls diverse cellular functions such as virulence and stress response [95]. A study has reported the establishment of a fast and efficient method for directly converting adenine to guanine in bacterial genomes. A systematic screening that targets the possibly editable adenine sites of *S. aureus* cntBC locates key residues for metal importation, demonstrating that the application of the system might greatly facilitate the bacterial genomic engineering [96].

## 3. Conclusions

During bacterial infection, essential nutrients, such as metal ions, are needed by the host in order to fight invading pathogens. The Gram-positive bacterium *S. aureus* can compete successfully with the host for iron and other metal ions. This is mainly achieved through the secretion of metal chelators, known as metallophores. Four siderophores (staphyloferrin A, staphyloferrin B, staphylobactin and Aureochelin) are synthesized and secreted by *S. aureus* to sequester iron from the extracellular environment. This bacterium has intake systems specific for these siderophores (ABC transporters), and it has the ability to uptake other microorganisms’ siderophores. Staphylopine is a nicotianamine-like wide spectrum metallophore produced by *S. aureus* as well. Staphylopine is synthesized by a three-step pathway and is exported and imported by specific membrane transporters. Siderophores are genetically regulated by Fur, while staphylopine is regulated by both Fur and Zur. The identification of the metal ion-acquiring systems utilized by *S. aureus* provides novel opportunities to distort this pathogen’s ability to compete with the host metal ions and thus minimize its production, enabling the development of new strategies to improve the therapeutic approaches.

## Figures and Tables

**Table 1:** Different types of metallophores produced by *S. aureus*.

Name	Type (Functional Group)	Molecular Formula	Molecular Weight (Da)	Specific for
Staphyloferrin A	Carboxylate (RCOOH)	C_17_H_24_N_2_O_14_	480	Iron
Staphyloferrin B	Carboxylate (RCOOH)	C_16_H_24_N_4_O_11_	448	Iron
Staphylobactin	Hydroxamate (R-CO-NH-OH)	unknown	822	Iron
Aureochelin	hydroxamate and catechol (R-CO-NH-OH and C_6_H_4_(OH)_2_)	unknown	577	Iron
Staphylopine	opine (amine and carboxylic acid)	C_13_H_19_N_4_O_6_^−^	327	broad-spectrum metallophore (nickel, zinc, cobalt, iron and copper)

**Table 2:** *S. aureus* metallophores regulation and transportation.

Name	Operon	Metal Uptake Regulator	Membrane Transporter
Staphyloferrin A	*sfaABCD*	Fur	HtsABC
Staphyloferrin B	*sbn (sbnABCDEFGHI)*	Fur	SirABC
Staphylobactin	*sbn*	Fur	SirABC
Staphylopine	*cnt (cntKLMABCDFE)*	Fur and Zur	CntB and CntC

## Data Availability

The study did not report any data.
